# [Corrigendum] miR‑26a inhibits ovarian cancer cell proliferation, migration and invasion by targeting TCF12

**DOI:** 10.3892/or.2024.8715

**Published:** 2024-02-15

**Authors:** Sainan Gao, Tingting Bian, Min Su, Yifei Liu, Yuquan Zhang

Oncol Rep 43: 368–374, 2020; DOI: 10.3892/or.2019.7417

Subsequently to the publication of the above paper, an interested reader drew to the authors' attention that, on p. 372, the data panels shown to represent the ‘NC’ and ‘Untreated’ data panels in Fig. 5A for the Transwell cell invasion assay experiments were apparently overlapping, suggesting that these data had been derived from the same original source, even though they were meant to have shown the results from differently performed experiments.

The authors were able to re-examine their original data files, and realize that this figure had been assembled incorrectly; the revised version of Fig. 5, now featuring the correct data panel for the ‘Untreated’ experiment, is shown on the next page. Note that the revisions made to this figure do not affect the overall conclusions reported in the paper. The authors are grateful to the Editor of *Oncology Reports* for allowing them the opportunity to publish this Corrigendum, and apologize to the readership for any inconvenience caused.

## Figures and Tables

**Figure 5.**
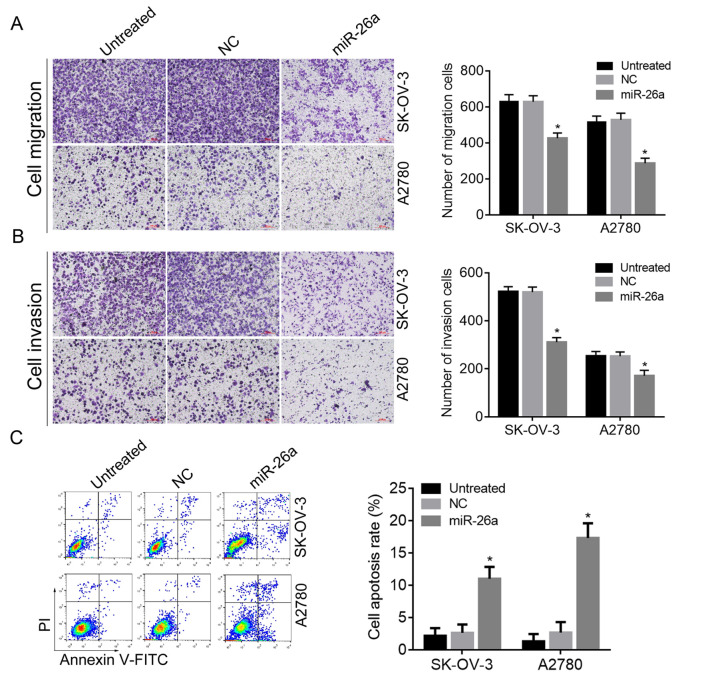
Effects of TCF12 inhibited by miR-26a on OC cell migration, invasion and apoptosis. (A) The migration of SK-OV-3 and A2780 cells was inhibited by miR-26a; (B) the invasion of SK-OV-3 and A2780 cells was inhibited by miR-26a; (C) apoptosis of SK-OV-3 and A2780 cells was induced by miR-26a. *P<0.05 compared with NC or untreated cells. TCF12, transcription factor 12; FITC, fluorescein isothiocyanate; PI, propidium iodide; NC, negative control. Scale bar, 100 µm.

